# Unusual Imaging Features of Dentigerous Cyst: A Case Report

**DOI:** 10.3390/dj7030076

**Published:** 2019-08-01

**Authors:** Carla Patrícia Martinelli-Kläy, Celso Ricardo Martinelli, Celso Martinelli, Henrique Roberto Macedo, Tommaso Lombardi

**Affiliations:** 1 Laboratory of Oral & Maxillofacial Pathology, Oral Medicine and Oral and Maxillofacial Pathology Unit, Division of Oral Maxillofacial Surgery, Department of Surgery, Geneva University Hospitals, University of Geneva, 1211 Geneva, Switzerland; 2 Centre for Diagnosis and Treatment of Oral Diseases, Ribeirão Preto 14025-250, Brazil

**Keywords:** dentigerous cyst, radiographic differential diagnosis, multislice computed tomography, hounsfield unit analysis

## Abstract

Dentigerous cysts (DC) are cystic lesions radiographically represented by a well-defined unilocular radiolucent area involving an impacted tooth crown. We present an unusual radiographic feature of dentigerous cyst related to the impacted mandibular right second molar, in a 16-year-old patient, which suggested an ameloblastoma or odontogenic keratocyst (OKC) because of its multilocular appearance seen on the panoramic radiography. A multi-slice computed tomography (MSCT), however, revealed a unilocular lesion without septations, with an attenuation coefficient from 3.9 to 22.9 HU suggesting a cystic lesion. Due to its extension, a marsupialization was performed together with the histopathological analysis of the fragment removed which suggested a dentigerous cyst. Nine months later, the lesion was reduced in size and then totally excised. The impacted mandibular right second molar was also extracted. Histopathological examination confirmed the diagnosis of a dentigerous cyst. One year later, the panoramic radiography showed a complete mandible bone healing. Large dentigerous cysts can sometimes suggest other more aggressive pathologies. Precise diagnosis is important to avoid mistakes since DC, OKC and ameloblastoma require different treatments. Histological examination is, therefore, essential to establish a definitive diagnosis. In our case, MSCT and the tissue attenuation coefficient analysis contributed to guide the diagnosis and management of the dentigerous cyst.

## 1. Introduction

A dentigerous cysts (DC) are defined as cystic lesions involving the crown of impacted teeth caused by fluid accumulation between the follicular epithelium and the crown of the tooth. It is considered the most common type of noninflammatory odontogenic cyst, and it occurs mainly in the lower third molar tooth of male patients with peak incidences in adolescents or young adults. Dentigerous cysts present a slow painless swelling and can cause the teeth displacement or teeth and bone resorption. Large cysts, however, may be associated with pain [1,2,3,4]. Histologically, they are represented by a cavity lined by the non-keratinizing thin epithelium without rete pegs. Their wall is usually fibrous and devoid of inflammatory cells [1].

Radiographically, DCs often present a unilocular radiolucent area around the crown of the impacted tooth surrounded by a well-defined sclerotic area [1,2,4,5]. Large cysts may cause cortical bone expansion. Moreover, DCs can rarely show a multilocular feature in the panoramic radiography. This is probably due to the cyst growth in areas of different bone densities [2]. In this case, the differential diagnosis should be made with other more aggressive lesions, such as ameloblastomas, odontogenic keratocysts (OKCs), other odontogenic tumours [1,2,3].

Computed tomography (CT), such as Cone Beam CT (CBCT) or multi-slice computed tomography (MSCT), has an important application in the evaluation of head and neck lesions. It is a non-invasive technique that permits the lesion size and margins, the bone destruction and expansion patterns to be precisely analysed [6,7]. In addition, MSCT provides an accurate measurement of the tissue attenuation coefficient [7,8,9,10]. Each tissue has the ability to absorb a certain proportion of X-rays as the bone, for example, which absorbs a lot of X-rays while the air almost none. An arbitrary scale named the Hounsfield scale shows these attenuation coefficients: −1000 Hounsfield unit (HU) represents the air attenuation, 0 is the water attenuation and +1000 HU is the bone attenuation [7,8].

This paper illustrates a case of an unusual dentigerous cyst initially diagnosed as ameloblastoma due to its multiloculated feature observed in panoramic radiography.

## 2. Case Presentation

**Figure 1:**
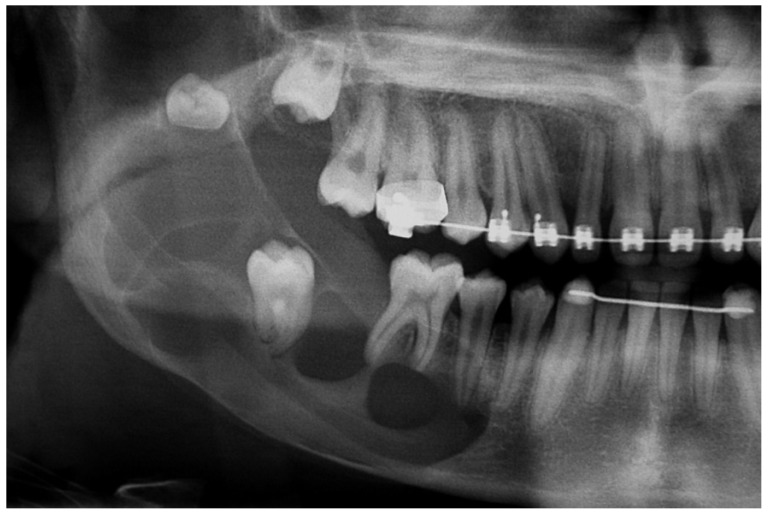
Panoramic radiography: A well-defined multiloculated radiolucent lesion involving impacted mandibular right second molar.

**Figure 2:**
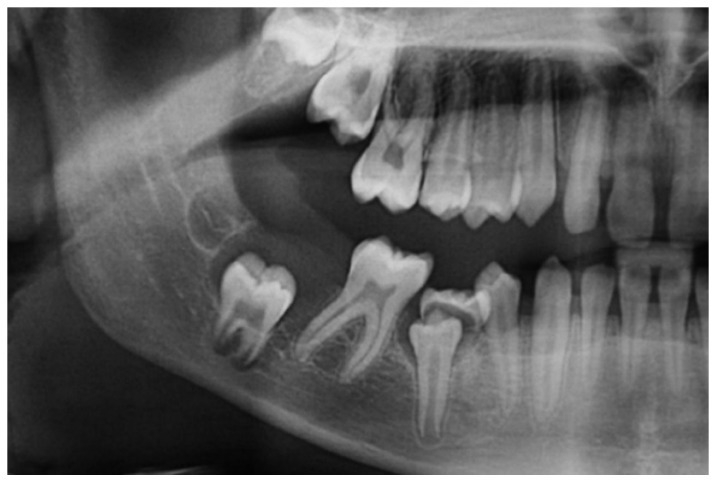
Panoramic radiography made at the age of thirteen: A unilocular well-defined radiolucent lesion (measuring around 4 mm) surrounding impacted mandibular right second molar.

A 16-year-old male was referred to the Centre for Diagnosis and Treatment of Oral Diseases with a clinical diagnosis of ameloblastoma having hemi-mandibulectomy as the suggested therapy. At admission, no relevant aspects within his medical history were observed. He brought a panoramic radiography (Figure 1) which revealed the presence of a multilocular lesion involving the impacted mandibular right second molar suggesting mainly an ameloblastoma or an OKC. It extended from the right mandibular notch to the lower right first premolar. A displacement of the mandibular canal and the lower right third molar towards the mandibular notch was also observed. We had access to another radiography taken at the age of thirteen, which presented a well-defined sclerotic area around the crown of the mandibular right second molar suggesting a hyperplastic dental follicle or a dentigerous cyst (Figure 2). During those three years, the patient did not consult. Intraoral examination revealed a slight painless swelling of the buccal and lingual cortical extending from the lower right first molar region to the mandibular branch (not shown).

**Figure 3:**
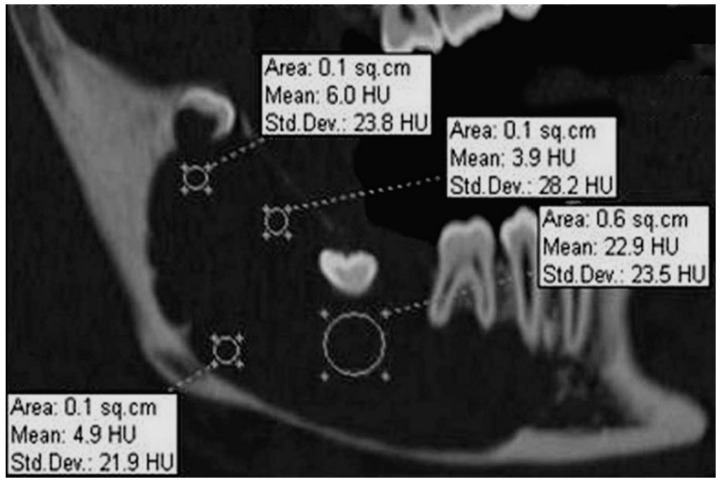
Sagital multi-slice computed tomography (MSCT) image: A large uniloculated lesion involving impacted mandibular right second molar. The lesion attenuation coefficient varies from 3.9 to 22.9 HU.

**Figure 4:**
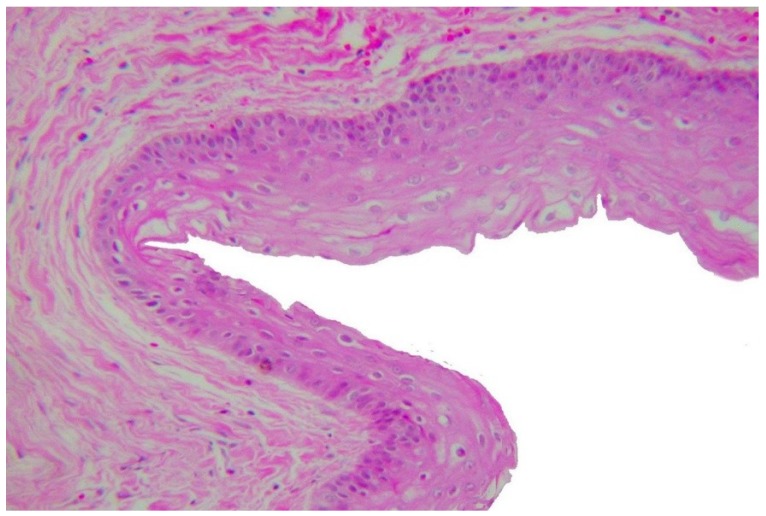
Photomicrograph: A non-keratinizing epithelial lining without rete pegs and a fibrous wall with rare inflammatory cells. HES, ×20.

**Figure 5:**
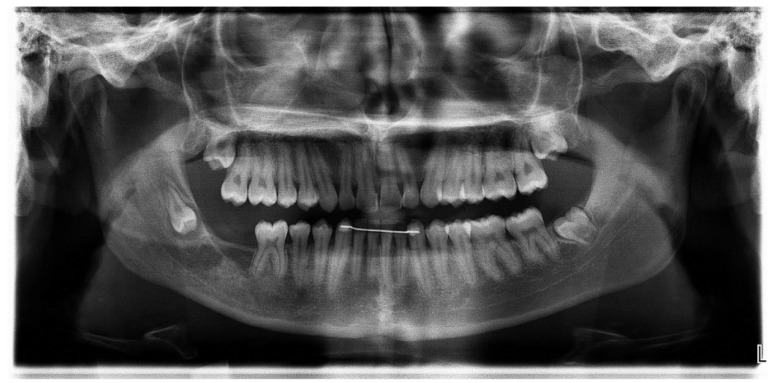
Panoramic radiography one year later: A complete mandible bone healing.

With the aim of further analysis of the lesion, an MSCT (Figure 3) was carried out, and unlike the panoramic radiography, it showed an area of hypoattenuation without septations. The Hounsfield unit (HU) value in the lesion varied from 3.9 to 22.9 HU, which suggested a lesion containing liquid that could be compatible with a dentigerous cyst or even a unicystic ameloblastoma. From this tissue attenuation coefficient analysis, a lesion such as an ameloblastoma (solid/multicystic type), or a cystic lesion containing a cheese-like substance as found in odontogenic keratocyst could be disregarded. Because of the lesion extension, marsupialization was performed together with the histopathological analysis of the fragment removed, which suggested a dentigerous cyst. During this procedure, a clear and pale fluid flowed out of the cavity. The lesion was irrigated weekly with chlorhexidine gluconate 0.12% oral solution. During treatment, a *candida* (*Candida* sp.) infection of the contents coming from the cavity (not shown) was detected through cytological examination and promptly treated with itraconazole capsules (1× daily for 10 days). After nine months, a considerable decrease of the lesion was observed on panoramic radiography (not shown). Excision of the cystic lesion, as well as extraction of the impacted mandibular right second molar, was then performed. Histopathological examination confirmed the diagnosis of a dentigerous cyst (Figure 4). One year later, a new panoramic radiography was carried out showing a complete mandible bone healing (Figure 5). The mandibular right third molar was only removed after its migration to the retromolar region in order to cause the least surgical trauma (Figure 5).

## 3. Discussion and Conclusions

Although dentigerous cysts often appear as a simple radiolucent area surrounding the crown of an impacted tooth, large cysts may show a multilocular feature and suggest other lesions such as ameloblastoma and OKC [2,3]. Therefore, a histological examination is essential in order to precise the diagnosis [1,11]. Dentigerous cysts present a cavity lined by a non-keratinized stratified epithelium containing between two and three layers of cuboidal and/or flattened cells. The connective tissue wall is usually fibrous and often devoid of inflammatory cells. Odontogenic keratocysts show a cavity lined by a thin and regular parakeratinized stratified squamous epithelium without rate pegs. The basal layer cells are cuboidal or columnar and are often hyperchromatic. The lumen of these cysts contains cheese-like material representing aggregated keratin scales. Similar to the dentigerous cyst, the interface between OKC’s epithelium and connective tissue is flat. Unicystic ameloblastomas are a variant of the ameloblastoma which appear as cysts. The epithelium lining these cysts, however, is composed of ameloblastic cells showing palisading and reverse nuclear polarity. The suprabasilar areas often display a loosen stellate reticulum appearance. The same histological features are found in the solid variant of ameloblastomas [1,2].

Unlike dentigerous cysts, ameloblastomas and odontogenic keratocysts have an aggressive behaviour and require different forms of treatment. Ameloblastomas are benign odontogenic tumours that usually require a wide surgical resection [2,12] whereas dentigerous cysts are usually treated with enucleation and curettage. Odontogenic keratocysts are benign cysts which have a relatively high rate of recurrence. They are preferably treated with surgical enucleation, marsupialization, decompression or marginal resection [13,14].

We presented a case of dentigerous cyst initially diagnosed as ameloblastoma having hemi-mandibulectomy as a suggested therapy. The ameloblastoma diagnosis was given by taking only the panoramic radiography and clinical aspect into account. The multilocular feature observed in the panoramic radiography is probably due to an uneven expansion of a large dentigerous cyst in areas of different bone densities [2]. It is known that panoramic radiography is a useful aid for diagnoses, but it only shows two-dimensional images of three-dimensional structures. In addition, it has a limited value to determine the lesion size and margins, tissue composition, as well as bone destruction and expansion patterns [15]. In our study, MSCT was carried out and, unlike the panoramic radiography, it showed an area of hypoattenuation without septations (Figure 3). We also found a variation of the tissue attenuation coefficient from 3.9 to 22.9 HU, which suggested a lesion containing serous fluid compatible with the content of a dentigerous cyst [16]. According to the literature, ameloblastomas (solid/multicystic type) have 35.9 plus/minus 12.6 HU and the OKCs show 28.4 plus/minus 10.5 [17] or up to 40 HU [14]. Unicystic ameloblastomas present a Hounsfield Unit of 31.0 plus/minus 6.0 HU [17]. It is known that MSCT provides accurate measurement of the tissue attenuation coefficient [7,8,9,10]. In addition, the incisional biopsy done during the marsupialization confirmed the diagnosis of dentigerous cyst [1,11]. 

Because of the lesion extension [11], the marsupialization was the initial treatment of choice. Decompression and marsupialization are both conservative procedures aimed at reducing the cyst size without damaging anatomical structures such as alveolar nerve, or even causing mandibular fracture during the surgery. In addition, these techniques allow a gradual apposition of bone tissue before the complete cyst enucleation [18,19,20,21]. 

Through cytological examination, *Candida* sp. infection coming from the cavity was observed and promptly treated with itraconazole (1× daily for 10 days). The cytological smear is used in clinical practice [22] and may be useful during the marsupialization treatment.

After a considerable decrease nine months later, the lesion was excised, and the impacted mandibular right second molar was extracted. According to Anavi et al. [19], the average of dentigerous cyst decompression is 7.6 months in patients under 18 years of age. In children, only the marsupialization, decompression and/or enucleation of the dentigerous cyst have been suggested in an attempt to preserve the tooth and promote its eruption [23,24,25]. In our case, preserving the tooth was not considered possible, mainly due to the size of the cystic lesion. Histological analysis confirmed the diagnosis of a dentigerous cyst which presented a wall of uninflamed fibrous connective tissue lined by a thin non-keratinized stratified epithelium.

In case of large or radiographically unusual cystic lesions, it is mandatory to perform a biopsy before planning surgical treatment. Depending on lesion size and diagnosis, an enucleation, marsupialization or decompression could be the forms of treatment chosen. In our case, the initial incisional biopsy made during the marsupialization process provided the diagnosis of a dentigerous cyst allowing a conservative approach. In addition, the tissue attenuation coefficient analysis done by MSCT was also useful in the diagnosis process to eliminate the possibility of other lesions such as a solid ameloblastoma or an odontogenic keratocyst.
